# Fate of Protocols Submitted to a French National Funding Scheme: A Cohort Study

**DOI:** 10.1371/journal.pone.0099561

**Published:** 2014-06-30

**Authors:** Evelyne Decullier, Laure Huot, François R. Chapuis

**Affiliations:** 1 Hospices Civils de Lyon, Pôle Information Médicale Evaluation Recherche, Unité de Recherche Clinique, Lyon, France; 2 Université de Lyon, RECIF, EAM Santé Individu Société 4128, Lyon, France; 3 Université Lyon 1, Lyon, France; Max Planck Society, Germany

## Abstract

**Background:**

The fate of clinical research projects funded by a grant has been investigated, but there is no information on the projects which did not receive funding. The fate of these projects is not known: do they apply for and/or receive funding from other sources or are they carried out without specific funding?

**Purpose:**

The aim of the study was to describe all clinical research projects submitted to a French national funding scheme (PHRC 2000) and to assess project initiation, completion and publication status taking into account whether or not they received funding.

**Methods:**

This study is a retrospective cohort. The initial project characteristics were retrieved from the submission files and follow-up information was collected from the primary investigator. The percentages of projects started, completed and published were studied.

**Results:**

A total of 481 projects were studied. Follow-up information was obtained for 366. Overall, 185 projects were initiated (51%); 139 of them were funded by the PHRC 2000 or other sources. The most commonly cited reason for not initiating a project was a lack of funding. Subsequently, 121 of the projects initiated were completed (65%). Accrual difficulties were the main reason cited to explain why studies were stopped prematurely or were still ongoing. Finally, 88 of the completed projects were published (73%). Amongst the completed projects, the only factor explaining publication was the statistical significance of the results.

**Conclusions:**

Obtainment of funding was a determining factor for project initiation. However, once initiated, the funding did not influence completion or publication.

## Introduction

According to good clinical practice, it is expected that all funded research will lead to publication. With this in mind, researchers have investigated the fate of funded studies. Of the 198 clinical trials funded by NIH and completed by 1988, 93% had been published 9 years later [1]. Another study on 1996 NIH grants found that each grant led to 7.6 manuscripts [2]. Moreover, the NIH requested in 2005 that all publications resulting from NIH-funded research be submitted to PubMed Central [3].

In 1992, a French funding scheme called the “Programme Hospitalier de Recherche Clinique” (PHRC) was created to develop clinical research [4]. This annual scheme is reserved for French hospitals. Each year, priority medical themes are defined by the French Ministry of Health and clinicians can apply for a grant for their projects. The PHRC is dedicated to funding clinical research studies from initiation to completion. A panel of national experts review and select projects to be funded by the French Ministry of Health. Each year, nearly 600 projects are submitted to the PHRC funding scheme. Overall, 30% of the projects submitted receive PHRC funding and in 2000, 17 million euros of funding was provided. Follow-up information is available for all funded projects [5]–[8], but there is no information on the projects which did not receive funding. In a French regional study, it was found that even projects rejected for a given source of funding resulted in publications [9]. The fate of rejected projects is not known: do they apply for and/or receive funding from other sources or are they carried out without specific funding?

The aim of this study was to describe the initial characteristics of, and to follow up on all projects submitted to the French national funding scheme PHRC in 2000, to assess project initiation, completion and publication status according to its source of funding.

## Methods

### Materials

All clinical research projects submitted for the PHRC 2000 funding scheme were included.

Each French tertiary teaching hospital has a research administration department which was invited to participate and to assign a research assistant to this study. All the research assistants attended a formal training session on abstracting study characteristics in January 2008 and on primary investigator survey in March 2008. They were locally responsible for collecting the initial project characteristics and obtaining follow-up data from the primary investigator for each project (either by interview or questionnaire).

### Study design

This is a retrospective cohort study conducted in 2 steps.

- Description of initial characteristics: For this step, each research assistant had to collect the data from the project file as submitted to the PHRC 2000 (planned number of patients, design, duration, and topic). Coding for topic and design was centralised (ED and LH).
- Follow-up of submitted projects: The primary investigator was contacted to obtain information on the source of funding (if received), the project’s initiation, the project’s completion and its publication status (in 2008–2010, i.e. 8 to 10 years after applying to the scheme). Some investigators might have submitted more than one project; however the statistical unit remained the project.

### Definition

The information on the obtainment of “PHRC 2000” funding was retrieved from the French Ministry of Health. This information was then completed by the data on other funding provided by the primary investigator (when available).

“PHRC 2000/other PHRC” was considered when the protocol obtained funding either from the PHRC 2000 itself or when the investigator declared they had obtained previous or subsequent PHRC funding (other PHRC).

”PHRC 2000/other PHRC/other funding” was considered when investigator obtained either the PHRC 2000 funding or PHRC funding in another year (other PHRC) or from a source of funding other than PHRC (other funding).

When the article reference was provided, we classified articles in 5 categories according to the percentiles of the impact factor of the journal [10]. In each medical specialty, 10% of journals are ranked A, 15% are ranked « B », 25% « C », 25% « D » and 25% « E ». We then attributed points to categories: 8 points for A; B = 6 points; C = 4 points; D = 3 points; E = 2 points; Not rated = 1 point, according to the conversion used by the French authorities as a publication indicator [11]. The publication quality score of the project was the number of points of the publication or the cumulative number of points when a project had more than one publication.

### Statistics

Descriptive analysis comprised of numbers and percentages. Percentages were compared using Chi^2^ statistics. For some questions, it was possible to choose more than one response; we reported the frequency of citations of the response by the number of projects concerned by the question.

The average number of publications was compared using the Mann-Whitney test. To investigate the time to publication, a survival analysis was performed on projects with sufficient information (publication dates available and consistent with the date of project initiation). A sensitivity analysis was also performed using questionnaire dates when the information on publication dates was missing.

The publication quality score was compared using the Mann-Whitney test.

### Ethical considerations

We conducted this study according to French law on epidemiological and descriptive studies [12]. No consent was needed as we retrieved no individual patient information.

## Results

Data was collected between January and December 2008; investigators survey was conducted between August 2008 and June 2010 and the centralised coding ended in January 2011.

### Characteristics of submitted projects

Four hundred and eighty-eight projects were submitted to the PHRC 2000 funding scheme. A total of 481 projects were studied (six files were lost and one was not a clinical research project). Of these, 101 received funding (21%). The initial characteristics are presented in table 1.

**Table 1:** Initial characteristics of protocols submitted to the PHRC 2000 funding scheme (n = 481).

Variable	Modality	n	%
Centres	Monocentre	145	30
	Multicentre	336	70
	*Regional*	*137*	*41*
	*National*	*173*	*51*
	*International*	*20*	*6*
	*Not provided*	*6*	*2*
Sample size provided	Yes	415	86
	No	66	14
*If provided, was it justified*	*Yes*	*254*	*61*
	*No*	*138*	*33*
	*Not applicable*	*23*	*6*
Inclusion period provided	Yes	239	50
	No	242	50
Topic*	Epidemiology	133	28
	Act/Gesture/Technique	74	16
	Strategy	64	13
	Drug	51	11
	Cell/gene therapy	43	9
	Practice evaluation	37	8
	Medical device	14	3
	Other	58	12
Study design*	Descriptive	100	21
	Analytic	135	28
	Experimental	239	50

*Information available for 474 studies.

### Primary investigators survey

Of the 481 projects studied, follow-up information was obtained for 366, giving a response rate of 76%. This response rate varied according to the obtainment of the PHRC 2000 grant (73% for non-funded projects and 86% for funded projects, p = 0.01). Amongst the protocols with follow-up data available, 24% were funded by the PHRC 2000 (n = 87), 10% were funded by other PHRC (n = 36) and 10% declared at least one other source of funding (n = 37) (table 2); for the remaining projects, no funding was declared (56%).

**Table 2:** Fate of protocols according to funding*.

		Overall	PHRC 2000		Chi^2^ p-value	PHRC 2000/other PHRC		Chi^2^ p-value	PHRC 2000/other PHRC/other funding		Chi^2^ p-value
			Funded	Not funded		Funded	Not funded		Funded	Not funded	
	Submitted	481	101	380		138	343		175	306	
Investigator response	Available	366	87 (86)	279 (73)		123 (89)	243 (71)		160 (91)	206 (67)	
	Not available	115	14 (14)	101 (27)		15 (11)	100 (29)		15 (9)	100 (33)	
Study initiated	No	172	9 (10)	163 (60)	<0.0001	15 (12)	157 (67)	<0.0001	20 (13)	152 (77)	<0.0001
	Yes	185	77 (90)	108 (40)		107 (88)	78 (33)		139 (87)	46 (23)	
	*Not provided*	*9*									
Study completed	No	64	23 (30)	41 (38)	0.28	31 (29)	33 (42)	0.06	43 (31)	21 (46)	0.08
	Yes	121	54 (70)	67 (62)		76 (71)	45 (58)		96 (69)	25 (54)	
Study published (of those initiated)	No	70	28 (37)	42 (39)	0.88	39 (37)	31 (40)	0.76	47 (34)	23 (50)	0.08
	Yes	113	47 (63)	66 (61)		66 (63)	47 (60)		90 (66)	23 (50)	
	*Not provided*	*2*									
Study published (of those completed)	No	31	14 (27)	17 (25)	1.00	19 (26)	12 (27)	1.00	23 (24)	8 (32)	0.45
	Yes	88	38 (73)	50 (75)		55 (74)	33 (73)		71 (76)	17 (68)	
	*Not provided*	*2*									

Values are n (%).

*Information on PHRC 2000 funding was obtained from the French Ministry of Health, information on other funding was provided by the primary investigator (327 out of 366 provided information).

### Project initiation

Overall, 51% (n = 185) of investigators declared that their project had started, 172 (47%) projects had not begun at the time of the survey and there was no information for nine projects. Project initiation was significantly related to the obtainment of PHRC 2000 funding: 90% of PHRC 2000 funded projects started *versus* 40% of non-funded projects (p<0.0001; table 2). The latter rate fell to 33% for projects without “PHRC 2000/other PHRC” funding and to 23% for projects without “PHRC 200/other PHRC/other Funding”. The most commonly cited reason for not initiating a project was a lack of funding followed by PHRC rejection.

### Project completion

Amongst the initiated projects, 121 (65%) were completed, 35 (19%) were stopped early and 29 (16%) were still ongoing at the time of the survey. Obtainment of funding was not related to the project’s final status (table 2). Concerning projects still ongoing and projects stopped early, the most frequently cited reason was accrual difficulties. These difficulties were declared to be related to a lack of patients and a lack of commitment from the investigators working on the research project.

### Publication

Publication status was available for 183 of the 185 initiated projects (table 2), 62% (n = 113) of initiated projects resulted in the publication of at least one scientific paper, representing 31% of submitted projects. When restricted to completed projects, 88 were published (74%). Two resulted in more than 25 papers and were excluded, as outliers, from further analyses.

The most cited reason explaining the non-publication of completed projects was that publication of the paper was in process, 5 investigators mentioned that the analysis had not yet been carried out. Three investigators cited the non-significance of results as a reason for non-publication and only one investigator cited rejection by the journal.

**Figure 1:**
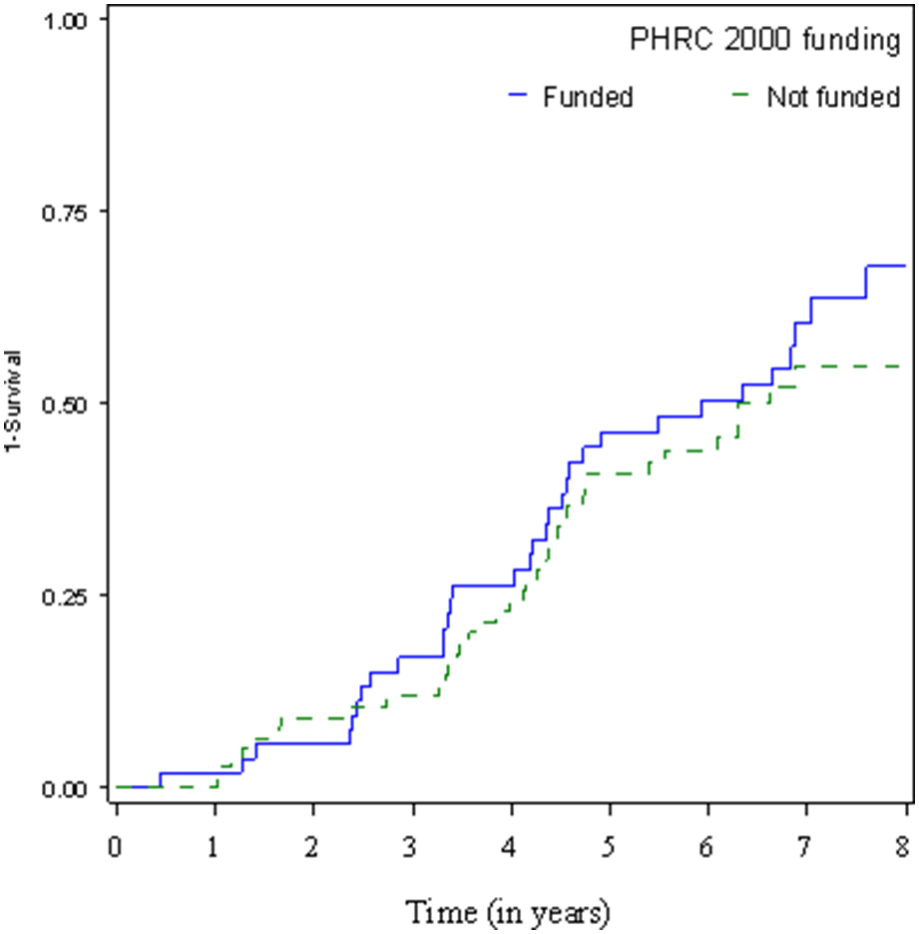
Time to publication.

The survival analysis was performed on 131 submitted projects with initiation and publication dates available. Median time to publication was 6.33 years (95% confidence interval: 4.74–7.59). There was no difference in time to publication for projects funded by the PHRC 2000 compared to other projects (figure 1, log-rank p-value = 0.38). When replacing missing publication, the analysis comprised 162 protocols; the median time to publication was 6.88 years (6.33–7.57).

Among the 111 projects with at least one publication (excluding the two outliers), the average number of publications was not significantly different according to the PHRC 2000 funding (2.9 for PHRC 2000 funded projects versus 2.8 for other projects; Mann-Whitney p-value = 0.67).

Concerning the quality of publication, complete references were available for 78 projects and the average publication quality score was not significantly different according to PHRC 2000 funding (14.24 for PHRC 2000 funded projects versus 12.16 for other projects; Mann-Whitney p-value = 0.88) nor PHRC 2000/other PHRC/other funding (13.79 for PHRC 2000/other PHRC/other funded projects versus 10.25 for non-funded projects; Mann-Whitney p-value = 0.60).

### Publication bias

When restricted to completed projects for which information on publication status and results was available (n = 94), the only explanatory variable for publication was the statistical significance of results. Publication rates were 90.5% (19/21) for studies with no hypothesis tested; 76.1% (51/67) for studies with a p-value of less than 0.05, and 33.3% (2/6) for studies with p-value greater than 0.05 (p = 0.02) (odds-ratio for p-values greater than 0.05 versus less than 0.05: 5.67 (95% CI: 0.96–33.61)).

## Discussion

In this study, we found that obtaining the French national public funding (PHRC 2000) or another source of funding was a determining factor for clinical research project initiation. However, once initiated, the funding did not influence project completion or publication.

This study is the first to propose the follow-up of projects after submission to a funding scheme regardless of their funded/non-funded status. The funding considered was a French national public funding scheme for the year 2000. We also considered the fate of projects according to the obtainment of other sources of funding, but the information on funding other than PHRC 2000 was based on the investigators’ declarations only.

We found that the rate of initiated studies was rather high, at least 87% when funding was obtained, and that even certain studies that did not receive funding were initiated. The rate of published studies was also high (73%) when compared to rates found from cohorts of protocols submitted to ethics committees in Europe (38% [13]), 31% [14]) or in the rest of the world (48% [15], 59% [16]) except for Dickersin (76% in a 1988 cohort [17]). It was similar to the rate found in a cohort of projects submitted for funding in a French region (67% [9]), but much lower to than the rate found for NIH funded studies (93% [1]) which was restricted to clinical trials. This could be explained by the fact that studies exploring cohorts of funded protocols usually find higher rates of publication than studies investigating protocols submitted to research ethics committees. In this study, we confirmed the existence of publication bias. However, the publication rate was high and only six investigators reported non significant p-values. Moreover, publication rates are also usually higher for studies with public as opposed to private funding. Publication rates and publication bias should therefore be interpreted carefully, taking into account the origin of the cohort and the presence and type of funding.

Concerning non-funded projects, a total of 46 protocols which did not obtain funding were initiated (23%) and 25 were completed. For projects without any declared source of funding, it is possible that leftover funds from other research or hospitals budgets might have been used; and one could question the reliability of a research undertaken without dedicated funding.

In conclusion, the French funding scheme, although it made it possible to initiate projects, did not increase the publication rate.
